# Midlife and old‐age cardiovascular risk factors, educational attainment, and cognition at 90‐years

**DOI:** 10.1002/alz.092144

**Published:** 2025-01-09

**Authors:** Anni Varjonen, Sari Aaltonen, Mia Urjansson, Teemu Palviainen, Toni T Saari, Jaakko Kaprio, Eero Vuoksimaa

**Affiliations:** ^1^ University of Helsinki, Helsinki Finland

## Abstract

**Background:**

Cardiovascular (CV) risk factors are associated with increased dementia risk, but their effects on cognition in the oldest‐old (90+) are not well studied. CV risk factors may appear to be protective against dementia due to reverse causation when they are measured during dementia process. To clarify this, we studied CV risk factors measured both at midlife and old age, with 48 years of follow‐up.

**Method:**

We had 96, 90‐97‐year‐olds from the NONAGINTA study consisting of population based older Finnish Twin Cohort study participants born in 1922‐1933. CV risk factors included blood pressure (BP), body mass index (BMI), and physical activity (PA) assessed via questionnaires in 1975, 1981 and 2021‐2023, and cholesterol in 2021‐2023. Telephone‐administered cognitive measures included semantic fluency (1‐minute animal naming), and immediate and delayed recall of three trial 10‐word list learning. We used linear and negative binomial regression analyses (delayed recall) with CV risk factors as independent variables (age, sex, education, and Apolipoprotein E genotype as covariates and adjusted for family data) and 90‐year‐old cognition as a dependent variable.

**Result:**

Those with 7‐11 years and ≥12 years of education had better immediate recall than those with ≤6 years of education (p = 0.003 & p<0.001). Those with ≥12 years (n = 19) of education also scored higher than those with ≤6 years of education both in semantic fluency and delayed recall (p<0.001). Higher middle‐age BMI was associated with better animal fluency (p = 0.011), but not with immediate or delayed recall. Old‐age BMI or cholesterol, and middle‐age or old‐age PA were not related to cognition. Those with high middle‐age BP (n = 6) scored higher in animal fluency (p = 0.005), immediate recall (p = 0.023) and delayed recall (p = 0.003) than those with normal BP. In contrast, those with high old‐age BP (n = 63) scored lower in animal fluency (P = 0.010), but not in immediate or delayed recall.

**Conclusion:**

The most robust finding was that higher (secondary) education is associated with better cognition even in late old‐age. Middle‐age high BP was associated with better old‐age cognition, but this relationship was reversed for old‐age BP. Paradoxical positive effect of high midlife BP may reflect earlier start of BP medication.

